# Effect of High-Intensity Interval Training on Rheumatoid Arthritis CD4+ T Cell Oxidative Metabolism

**DOI:** 10.1093/geroni/igab046.3249

**Published:** 2021-12-17

**Authors:** Brian Andonian, David Bartlett, Nancie MacIver, William Kraus, Kim Huffman

**Affiliations:** 1 Duke University School of Medicine, Durham, North Carolina, United States; 2 Duke University, Durham, North Carolina, United States

## Abstract

Persons with rheumatoid arthritis (RA) have poor cardiorespiratory fitness and accelerated biological aging driven by systemic impairments in metabolism and inflammation. In this study of older RA participants, our goal was to identify the effects of a high-intensity interval training (HIIT) program on cardiorespiratory fitness and peripheral CD4+ T cell metabolism. We isolated CD4+ T cells from peripheral blood mononuclear cells in sedentary female RA participants (n=6; age=64.0+/-6.3 years) who underwent cardiopulmonary exercise testing and phlebotomy before and after 10 weeks of HIIT. HIIT improved RA cardiorespiratory fitness by 6.5+/-6.0% (pre-HIIT VO2 peak=25.1+/-5.1 ml/kg/min, post-HIIT VO2 peak=26.7+/-5.0; p=0.05). As measured by Seahorse XF Mito Stress Test, there were no significant mean changes in CD4+ T cell oxidative (oxygen consumption rate (OCR); pmol/min) or glycolytic (extracellular acidification rate (ECAR); mpH/min) metabolism, however there was large interindividual variability. RA peripheral CD4+ T cells preferred glycolytic metabolism (pre-HIIT mean basal OCR/ECAR ratio=0.78+/-0.13 pmol/mpH), while HIIT non-significantly shifted cellular preference toward oxidative metabolism (post-HIIT mean basal OCR/ECAR ratio=0.86+/-0.16; p=0.30). Increases in RA cardiorespiratory fitness following HIIT were significantly associated with increases in RA peripheral CD4+ T cell OCR/ECAR ratio (Spearman’s rho=1.0, p<0.001) and basal and maximal respiration (rho=0.89, p=0.02 for both). Additionally, increases in CD4+ T cell mitochondrial ATP-linked respiration were significantly associated with increased quantities of circulating naïve CD4+CCR7+CD45RA+ T cells (rho=0.89, p=0.02). Our findings suggest that targeting cardiorespiratory fitness may be key in modulating T cell specific oxidative metabolism and function to prevent immunosenescence in older patients with chronic inflammatory diseases.

